# Intelligent Reflecting Surfaces Beamforming Optimization with Statistical Channel Knowledge

**DOI:** 10.3390/s22062390

**Published:** 2022-03-20

**Authors:** Victoria Dala Pegorara Souto, Richard Demo Souza, Bartolomeu F. Uchôa-Filho, Yonghui Li

**Affiliations:** 1 Center of Social and Technological Sciences, Catholic University of Pelotas, Pelotas 96015-560, RS, Brazil; 2 Department of Electrical and Electronic Engineering, Federal University of Santa Catarina, Florianópolis 88040-900, SC, Brazil; richard.demo@ufsc.br (R.D.S.); uchoa@eel.ufsc.br (B.F.U.-F.); 3 School of Electrical and Information Engineering, University of Sydney, Sydney, NSW 2006, Australia; yonghui.li@sydney.edu.au

**Keywords:** intelligent reflecting surfaces, beamforming, statistical channel station information, genetic algorithms

## Abstract

Intelligent Reflecting Surfaces (IRSs) are emerging as an effective technology capable of improving the spectral and energy efficiency of future wireless networks. The proposed scenario consists of a multi-antenna base station and a single-antenna user that is assisted by an IRS. The large number of reflecting elements at the IRS and its passive operation represent an important challenge in the acquisition of the instantaneous channel state information (I-CSI) of all links as it adds a very high overhead to the system and requires equipping the IRS with radio-frequency chains. To overcome this problem, a new approach is proposed in order to optimize beamforming at the BS and the phase shifts at the IRS without considering any knowledge of I-CSI but while only exploring the statistical channel state information (S-CSI). We aim at maximizing the user-achievable rate subject to a maximum transmit power constraint. To achieve this goal, we propose a new two-phase framework. In the first phase, both the beamforming at the BS and IRS are designed based only on S-CSI and, in the second phase, the previously designed beamforming pair is used as an initial solution, and beamforming at the BS and IRS is designed only by considering the feedback of the SNR at UE. Moreover, for each phase, we propose new methods based on Genetic Algorithms. Results show that the developed algorithms can approach beamforming with I-CSI but with significantly reduced channel estimation overhead.

## 1. Introduction

Intelligent reflecting surfaces (IRS), also called reconfigurable intelligent surfaces [1], and software-controlled metasurfaces [2], have recently attracted great interest. An IRS is a meta-surface comprising a large number of reflecting elements, capable of reflecting the incident signal with a given phase/amplitude shift. By densely deploying IRS in wireless communication networks and intelligently coordinating their elements, the wireless channels between the transmitter and receiver can be intentionally and deterministically controlled to improve signal quality at the receiver and the network’s capacity [3,4,5,6].

The optimization of the beamforming at the IRS and the BS has been studied in some works [3,5,7,8,9,10,11,12,13,14]. In [5], energy efficiency is maximized by jointly optimizing beamforming at the IRS and the power allocation. In [8,9], the authors design beamforming at the BS and IRS with the goal of minimizing BS’ transmit power. In [15], a system using IRS and orthogonal frequency division multiplexing is proposed for maximizing the achievable rate. The authors optimize beamforming at IRS and BS power. The works in [8,9,15] consider continuous phases at the IRS, while those in [3,11,13,14] make the more practical assumption of discrete phases. Moreover, in [12], the phase shift model includes a phase-dependent amplitude variation in IRS element reflection coefficients. The above-mentioned works consider perfect knowledge of the instantaneous channel state information (I-CSI) at the BS, which is very difficult to obtain in practice due to the large number of elements at the IRS and  would need the deployment of RF chains at the IRS. Moreover, recently, in [7], we investigated the joint optimization of BS and IRS beamforming with the objective of minimizing the BS transmit power without any prior CSI knowledge. In addition, in [7], we propose a new method based on the Particle Swarm Optimization (PSO) technique. As in [8,9,15], the model in [7] assumes continuous phases at the IRS, which is not feasible in practice due to hardware limitations.

In order to overcome this issue, recently, some works started exploring the statistical channel state information (S-CSI) [15,16,17,18,19,20,21,22,23,24,25], which is easier to estimate. Accordingly, in [15,16,17,18], the authors designed phase shifts at the IRS by considering only S-CSI in order to improve system performance. However, in those works, the design of beamforming at the BS is not considered, which simplifies the optimization problem and, consequently, reduces system performance. In [19], the authors consider only S-CSI to jointly design beamforming at the BS and at the IRS in order to maximize the ergodic sum capacity. In [20,21], a new two-timescale transmission protocol was proposed to maximize the achievable average sum-rate for an IRS-aided multi-user system. Specifically, the IRS phase shifts are first optimized only based on the S-CSI of all links. Then, the transmit beamforming vector at the base station (BS) is designed based on the I-CSI of the users’ effective channels. A multi-user extension for [20,21] is proposed in [22] and the average transmit power minimization problem is studied. To finish, some works also study the beamforming design at the IRS by considering only random rotations schemes without the need of I-CSI knowledge at the IRS [26,27,28], however, considering only random phase shifts at the IRS limits system performance, and to design beamforming at BS, it is still necessary to consider I-CSI knowledge at the BS [27], which is a challenge in practical scenarios.

Currently, evolutionary computation techniques have attracted great interest due to their ability to solve complex optimization problems from different fields. These techniques are inspired by biological evolution process, swarm behavior, and physics [29]. Among the main evolutionary computation techniques, Genetic Algorithms (GA) and PSO are algorithms widely used for handling complex optimization problems [7,23,24,25,30]. Although both GA and PSO are able to successfully solve different complex problems, GA is discrete by nature, i.e., it is preferable to use GA to solve discrete optimization problems. In contrast, PSO is suited for continuous optimization problems. PSO needs to be deeply modified to solve discrete optimization problems and usually does not present great performance for complex problems of such forms.

Therefore, as continuous phases at the IRS are not feasible in practice, usually GA is the most suitable heuristic method to design phase shifts at the IRS. From this, some works started to explore this technique to design phase shifts at the IRS by considering S-CSI knowledge [23,24,25]. More specifically, a multi-pair communication system assisted by an IRS is considered in [23], while a method based on GA was proposed in order to maximize the system’s achievable rate by solely optimizing discrete or continuous phase shifts at IRS by considering only the knowledge of S-CSI. In addition, in [24], system performance in terms of the achievable rate for a massive MIMO (mMIMO) system assisted by an IRS is studied. The authors in [24] designed phase shifts at the IRS by only considering S-CSI knowledge and proposed a novel GA to maximize the sum rate and the minimum user rate. In addition, the work in [25] considers the performance of an IRS-aided MIMO system, and the design of the phase shifts at the IRS elements was proposed based on S-CSI. The authors present a closed-form expression for the uplink ergodic data rate and, based on this, they consider the GA proposed in [24] to solve the sum-rate maximization problem. Although, the works in [24,25] consider the design of the phase shifts at the IRS with S-CSI only, the maximum ratio combining (MRC) is utilized to design beamforming at BS and, consequently, the knowledge of the instantaneous CSI at the BS is necessary, which is very difficult to obtain in practice. In addition, in [19], although the authors do not consider any I-CSI knowledge, beamforming at the BS and at the IRS are designed based only on S-CSI, limiting system performance, while they do not explore the full potential gains at the BS and at the IRS.

### Contributions

Motivated by the above challenges, in this paper, we consider a MISO (Multiple-Input Single-Output) communication system aided by an IRS. Then, we propose a new framework split into two phases in order to design beamforming at the IRS and BS by considering new methods based on the main GA features. We aim to maximize the system achievable rate while meeting a maximum transmit power requirement at the BS through beamforming optimization at IRS and BS. To achieve the main goal of this work, first, beamforming at the BS and the phase shifts at the IRS are designed based only on S-CSI and, secondly, such beamforming pairs are used as an initial solution of the proposed method in order to improve its convergence; the beamforming at BS and IRS are designed based only on the feedback of SNR at UE. Here, it is important to note that the proposed solution does not require any explicit channel estimation process. As we can observe, differently from [15,16,17,18,23,24,25], both beamforming at the BS and the phase shifts at the IRS are designed, which considerably increased the complexity of the optimization problem. In addition, the methods proposed differ significantly, in terms of GA implementation, from those in [23,24,25], as we consider a more specialized algorithm design. Moreover, different from [20,21,22,24,25], the proposed solution does not consider any knowledge of I-CSI. In addition, different from [26,27,28], where the beamforming at IRS was designed based on random phase shifts at the IRS, in this paper, we designed a suboptimal beamforming vector at IRS, which allows the better exploration of IRS gain. In addition, in [19], although the authors do not consider any I-CSI knowledge, beamforming at the BS and at the IRS is designed based only on S-CSI, without any other further refinements based on SNR feedback as in this work. Finally, different from [3,5,7,8,9,10,11,12,13,14], we consider discrete phases at the IRS elements. Therefore, the main contributions of this work are as follows:

1. We develop a novel framework based on meta-heuristic methods to design beamforming at BS and IRS without requiring I-CSI;
2. We reveal that the proposed solution can achieve a close-to-ideal performance by considering only S-CSI knowledge with low training overhead.

The rest of this paper is organized as follows. Section 2 presents the system model and the optimization problem. Section 3 describes GA operation and its principles. Section 4 reports the proposed approach. Section 5 presents simulation results. Finally, Section 6 concludes the paper.

  *Notations:* An Italic letter such as *a* denotes a variable, bold-faced upper case letters such as **A** denote a matrix, and a bold-faced lower case letter **a** represents a vector, where [***a***]${}_{i}$ is the *i*-th element of **a**. In addition, ${\left(\cdot \right)}^{H}$ denotes the conjugate transpose.

## 2. System Model

**Figure 1:**
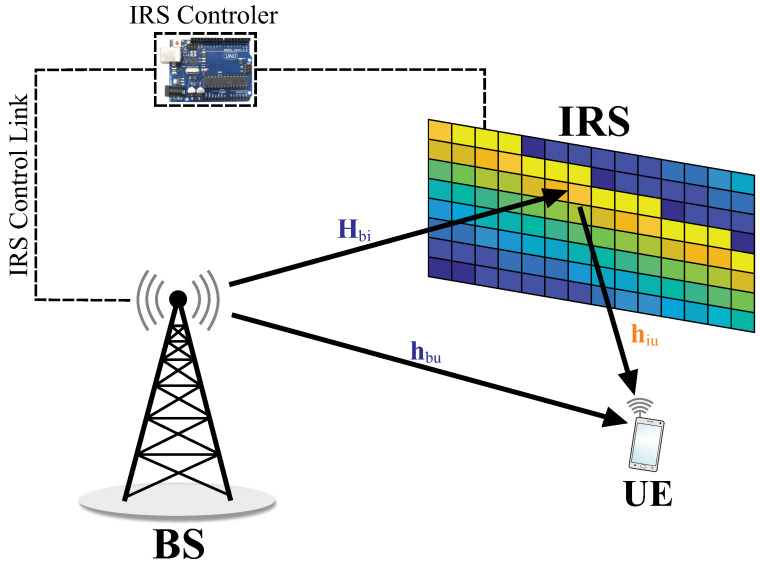
An IRS-assisted single-user downlink communication system.

We consider a wireless communication system where an IRS is deployed to assist communication between a multiple-antenna BS and a single-antenna user equipment (UE), as shown in Figure 1. The BS is assumed to be equipped with *N* antennas and IRS is equipped with $M=M_{h}\;\times \;M_{v}$ reflecting elements. In accordance to [31], in this paper, we consider that the channels change slowly, as we assume limited mobility.

The received signal at UE is as follows [9]:

(1) $$y=\left(\sqrt{\beta_{c}}h_{\mathrm{iu}}^{H}\theta H_{\mathrm{bi}}+\sqrt{\beta_{d}}h_{\mathrm{bu}}^{H}\right)ws+n,$$

where $\beta_{d}$ and $\beta_{c}$ are the pathloss of the direct link (BS-UE) and the cascade link (BS-IRS-UE), respectively. $h_{\mathrm{iu}}^{H}\in C^{1\times M}$ is the IRS-UE channel vector, $H_{\mathrm{bi}}\in C^{M\times N}$ is the BS-IRS channel matrix, $h_{\mathrm{bu}}^{H}\in C^{1\times N}$ is the BS-UE channel vector, $w\in C^{N\times 1}$ is the beamforming vector at the BS, *s* denotes the transmitted data defined as an independent random variable with zero mean and unit variance, and $n∼\mathrm{CN}(0,\sigma^{2})$ is the additive white Gaussian noise. In addition, $\Theta =\mathrm{diag}\left({\left[\xi \right]}_{1}e^{j{\left[\theta \right]}_{1}},\cdots ,{\left[\xi \right]}_{M}e^{j{\left[\theta \right]}_{M}}\right)$ where ${\left[\xi \right]}_{m}\in \left[0,1\right]$ (with m=1,⋯,M) and $\theta =\left[{\left[\theta \right]}_{1},\cdots ,{\left[\theta \right]}_{M}\right]$ denote the amplitude reflection coefficient and the discrete phase shift vector at the IRS, respectively. The discrete phase ${\left[\theta \right]}_{m}$ takes values on the set $T=\left\{0,\frac{2\pi }{K},\cdots ,\frac{2\pi (K-1)}{K}\right\}$, where $K=2^{B}$, and *B* denotes the number of bits per each element at the IRS. Moreover, in accordance to [8,9], we consider ${\left[\xi \right]}_{m}=1$ for m=1,⋯,M.

In this paper, we consider two different pathloss models; i.e., for the BS-UE direct link, pathloss (in dB) is provided by the following [32]:

(2) $$\beta_{d}=G_{t}+G_{r}-22\log_{10}\left(d_{\mathrm{BU}}\right)-\beta_{0},$$

where $G_{t}$ and $G_{r}$ are the antenna gain at the BS and UE (in dBi), respectively, $d_{\mathrm{BU}}$ is the distance between the BS and UE, and $\beta_{0}$ is a constant path loss factor. The pathloss of the BS-IRS-UE compound link (in dB) is given by the following [33]:

(3) $$\beta_{c}=G_{t}+G_{r}+10\log_{10}\left(D\right)-\beta_{1},$$

where $\beta_{1}$ is a constant path loss factor, $D={\left(\frac{ab}{d_{\mathrm{BI}}d_{\mathrm{IU}}}\right)}^{2}\cos^{2}\phi_{c}$, $d_{\mathrm{BI}}$ is the distance between the BS and IRS, $d_{\mathrm{IU}}$ denotes the distance between the IRS and UE, $a=M_{h}d_{\mathrm{IRS}}\lambda$ and $b=M_{v}d_{\mathrm{IRS}}\lambda$ are the IRS dimensions, respectively, λ denotes the wavelength, and $\phi_{c}=\arctan\left(\frac{y_{b}}{x_{b}}\right)$ denotes the angle of arrival to the IRS, where $\left(x_{b},y_{b}\right)$ denotes BS position [33].

In addition, to compute the small-scale fading, we consider the Rician fading channel model for BS-UE and IRS-UE links, which are given by the following:

(4) $$h_{i}=\left(\sqrt{\frac{\kappa_{i}}{1+\kappa_{i}}}h_{i}^{\mathrm{LoS}}+\sqrt{\frac{1}{1+\kappa_{i}}}h_{i}^{\mathrm{NLoS}}\right),$$

where i∈{iu,bu}, which represents the IRS-UE and BS-UE link, respectively, and $\kappa_{i}$ is the Rician factor. For the sake of simplicity, in this work, we consider $\kappa_{i}=\kappa \;\forall \;i$. Moreover, $h_{i}^{\mathrm{LoS}}$ represents deterministic LoS components, which define S-CSI, and $h_{i}^{\mathrm{NLoS}}$ denotes the Rayleigh fading of the links. It is important to underline that, in accordance to [31,34,35,36], we consider an LoS link between BS and IRS $\left(H_{\mathrm{bi}}=H_{\mathrm{bi}}^{\mathrm{LoS}}\right)$, which is a reasonable assumption in practical scenarios. In addition, we consider partial LoS links between BS and UE (BS-UE link), and between IRS and UE (IRS-UE link), both defined by (4), which is a general formulation and is in accordance to [8,15]. Therefore, in this work, we define S-CSI as $\left(H_{\mathrm{bi}}^{\mathrm{LoS}},h_{\mathrm{bu}}^{\mathrm{LoS}},h_{\mathrm{iu}}^{\mathrm{LoS}}\right)$. Since we consider low-mobility users,  S-CSI changes slowly.

The LoS components of all links are expressed by the antenna array responses at the IRS and at the BS, which are dependent on the array geometry. In this work, we consider that the BS is equipped with a Uniform Linear Array (ULA) as in [16,37] and IRS is equipped with a Uniform Planar Array (UPA); thus, the LoS components of the BS-IRS, BS-UE, and IRS-UE links are, respectively, given by $H_{\mathrm{bi}}^{\mathrm{LoS}}=a_{\mathrm{IRS}}{\left(\varphi_{\mathrm{IRS}},\theta_{IRS}\right)}^{H}a_{\mathrm{BS}}\left(\theta_{\mathrm{BS}}\right)e^{j\phi_{\mathrm{bi}}}$, $h_{\mathrm{bu}}^{\mathrm{LoS}}=a_{\mathrm{BS}}\left(\theta_{\mathrm{BS}}\right)e^{j\phi_{\mathrm{bu}}}$, and $h_{\mathrm{iu}}^{\mathrm{LoS}}=a_{\mathrm{IRS}}\left(\varphi_{\mathrm{IRS}},\theta_{IRS}\right)e^{j\phi_{\mathrm{iu}}}$, where $\varphi_{\mathrm{IRS}}$ and ($\theta_{\mathrm{IRS}}$, $\theta_{\mathrm{BS}}$) are azimuth and elevation angles, respectively, and $\phi_{\mathrm{bi}}$, $\phi_{\mathrm{bu}}$, and $\phi_{\mathrm{iu}}$ denote the random phase in the LoS components of the BS-IRS, BS-UE, and IRS-UE links, respectively. $a_{\mathrm{BS}}\left(\theta_{\mathrm{BS}}\right)$ and $a_{\mathrm{IRS}}\left(\varphi_{\mathrm{IRS}},\theta_{IRS}\right)$ denote the ULA response and UPA response, respectively, and are given by the following [1]:

(5) $$a_{\mathrm{BS}}\left(\theta_{\mathrm{BS}}\right)=\frac{1}{N}\left[1,e^{jkd_{\mathrm{BS}}\cos\left(\theta_{\mathrm{BS}}\right)},\cdots ,e^{jkd_{\mathrm{BS}}\left(N-1\right)\cos\left(\theta_{\mathrm{BS}}\right)}\right],$$

(6) $$a_{\mathrm{IRS}}\left(\varphi_{\mathrm{IRS}},\theta_{IRS}\right)=\left[e^{j\Lambda {\left(\varphi_{\mathrm{IRS}},\theta_{\mathrm{IRS}}\right)}^{T}u_{1}},\cdots ,e^{j\Lambda {\left(\varphi_{\mathrm{IRS}},\theta_{\mathrm{IRS}}\right)}^{T}u_{N}}\right],$$

where $k=\frac{2\pi }{\lambda }$ is the wavenumber, $u_{m}=\left[0,I\left(m\right)d_{\mathrm{IRS}}\lambda ,J\left(m\right)d_{\mathrm{IRS}}\lambda \right]$ for m=1,⋯,M, where $I\left(m\right)=\;\mathrm{mod}\;\left(m-1,M_{h}\right)$, $J\left(m\right)=\lfloor \left(m-1\right)/M_{h}\rfloor$, $d_{\mathrm{BS}}$, and $d_{\mathrm{IRS}}$ are the distances among the BS elements and among the IRS elements, respectively, and $\Lambda \left(\varphi_{\mathrm{IRS}},\theta_{\mathrm{IRS}}\right)=\frac{2\pi }{\lambda }{\left[\cos\left(\varphi_{\mathrm{IRS}}\right)\cos\left(\theta_{\mathrm{IRS}}\right),\sin\left(\varphi_{\mathrm{IRS}}\right)\cos\left(\theta_{\mathrm{IRS}}\right),\sin\left(\theta_{\mathrm{IRS}}\right)\right]}^{T}$ is the wave vector [38]. Finally, the achievable rate in bps/Hz at the UE is as follows:

(7) $$R=\log_{2}\left(1+\mathrm{SNR}\right),$$

where SNR is the signal-to-noise ratio at the UE, and it can be written as follows.

(8) $$\mathrm{SNR}=\frac{|\left(\sqrt{\beta_{c}}h_{\mathrm{iu}}^{H}\theta H_{\mathrm{bi}}+\sqrt{\beta_{d}}h_{\mathrm{bu}}^{H}\right)w{|}^{2}}{\sigma^{2}}.$$

### Optimization Problem

In this work, we aim to optimize beamforming at BS and the phase shifts at IRS in order to maximize the achievable rate at the user by considering a maximum transmit power constraint. This optimization problem is provided by the following:

(9) $$\begin{array}{cccc} \; & \underset{w,\theta }{\mathrm{Maximize}}\; & \; & R\\ \; & \mathrm{Subject}\;\mathrm{to}\; & \; & {\left||w|\right|}^{2}\leq P_{t},\\ \; & \; & & {\left[\theta \right]}_{m}\in T,\;m=1,\cdots ,M, \end{array}$$

where $P_{t}$ is the transmit power at BS. From the constraints in (9), it can be seen that the optimization problem is non-convex and cannot be solved by a standard method. Accordingly, to overcome this problem, we propose a novel solution based on GA described in Section 4.

## 3. Genetic Algorithms

The concept of GAs is based on the Theory of Evolution by natural selection, as proposed by Charles Darwin [39], in which individuals change over time and these changes help them to survive and to reproduce in order to generate descendants in the next generations. GAs are known to have simple implementation and to deliver good results in many optimization problems [23,40]. The terms typically used in GA come from biology, and for facilitating understanding, Table 1 lists the main terms used in this work and their meaning in the proposed optimization problem. Moreover, the operation of a GA is summarized next [41,42]:

1. Randomly generate the individuals of the first generation;
2. Compute the fitness of each individual;
3. **Elitism:** Create a set with some of the fittest individuals of the current generation and perpetuate these individuals to the next generation;
4. Select some individuals (named parents) by the Tournament Method (*Selection Method*), which will be submitted to the Reproduction Process (*Crossover and Mutation Operators*).
5. **Crossover Operator:** This operator makes the permutation of the genetic material of the selected parents and generates children with probability $p_{c}$, generating a set of new individuals.
6. **Mutation Operator**: The individuals generated in the previous step are submitted to the *Mutation Operator* with probability $p_{\mathrm{mut}}$, generating another set of individuals.
7. Generate the new population by the union of the sets of individuals generated in Steps 3 and 6.
8. The GA is completed if the stop criterion is fulfilled. Otherwise, return to Step 2.

In order to better explain the operation of a GA, the main methods and operators used in this optimization technique are explained next.

### 3.1. Selection Method

In GA, selection methods are used to choose individuals for participating in the reproduction process. Several selection methods exist in the literature [42]. In the present work, we consider the Tournament Method [42] by which $T_{\mathrm{tourn}}$ randomly chosen individuals enter a tournament; the winning individual, i.e.,  the one with the highest fitness, is selected [42,43]. This process is repeated until the required number of individuals is reached. The selected individuals are submitted to the reproduction process.

### 3.2. Reproduction Process

The reproduction process basically consists of crossover and mutation operators, which are applied in the individual selected by the selection method [41,42,43]. The basic operation of these operators are described next.

#### 3.2.1. Crossover Operator

From this operator, the selected individuals (named parents) have their features permuted to form descendants. In this work, we implemented the *Uniform Crossover Operator* for real coding [42], for which its basic operation is described next. This operator follows the crossover probability, which directly influences the diversity of the next generation. Thus, for a given crossover probability ($p_{c}$), the selected individuals are submitted to the crossover operator, i.e., a chain of bits (0 or 1) is randomly generated. For each bit, if the value is “1” (respectively, “0”), then the current gene of the first child receives the current gene of the first (respectively, second) parent and the current gene of the second child receives the current gene of the second (respectively, first) parent. Otherwise, for a given probability ($1-p_{c}$), the selected parents are perpetuated for the next generation.

#### 3.2.2. Mutation Operator

The Mutation Operator is applied on individuals resulting from the crossover operator in order to add new features in individuals and increase their genetic diversity. This operator aims at reducing the likelihood that the GA becomes stuck at a local optimum. This operator follows a given mutation probability ($p_{\mathrm{mut}}$) [42,43]. In this paper, we implemented the *Real Random Mutation Operator*. This operator randomly selects a new value from the set of valid values of that optimization variable to replace the current one of the selected gene.

### 3.3. Elitism

Elitism is a method that is used to ensure that the genetic material of the fittest individuals of the current generation will be perpetuated to the next generation, improving GA convergence. In Elitism, the best individuals of a population are selected and kept for the next generation without experiencing crossover and mutation operators. This method guarantees that the quality of the solution obtained by the GA does not decrease in the next generation. In addition, Elitism improves the performance of GA because this concept ensures that the GA does not waste time rediscovering good solutions that are previously analyzed [42,44].

## 4. Proposed Solution

**Figure 2:**
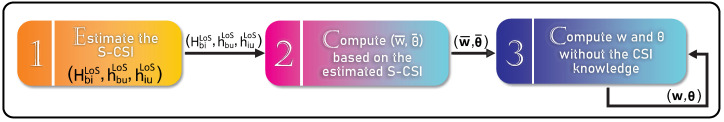
Diagram of the proposed solution.

In this paper, a solution based on GA is proposed to solve the optimization problem in (9). In the proposed approach, we exploit S-CSI in the beginning of the beamforming design process. We divide our proposed solution in three phases, as illustrated in Figure 2. In the first phase, S-CSI is acquired using standard estimation techniques [45,46] and fed back to BS (the acquisition of the S-CSI is left for future work, here, we focus on analyzing the influence of the S-CSI knowledge on the optimization problem.) In the second phase, considering only S-CSI, the beamforming vectors at BS and IRS are determined at BS and the suboptimal beamforming pair, evaluated in terms of the instantaneous achievable rate in (7), is computed. It is important to emphasize that, in this phase, we only consider the LoS components of all channel links. Thus, for each beamforming pair, BS receives the feedback of the SNR at UE, and the ongoing beamforming pair is evaluated based on (7). Therefore, we maximize the instantaneous achievable rate. This process is performed for all pairs. Note that, in this phase, I-CSI does not need to be explicitly estimated; therefore, we do not require any instantaneous channel estimation process, which considerably reduces the training overhead. To finish, the main phases are described below.

1. **Estimate S-CSI**: In this phase, we consider that IRS is in the sensing mode and the S-CSI of all links $\left(H_{\mathrm{bi}}^{\mathrm{LoS}},h_{\mathrm{bu}}^{\mathrm{LoS}},h_{\mathrm{iu}}^{\mathrm{LoS}}\right)$ is estimated by considering dedicated sensors/receiving circuits at the IRS using standard estimation techniques [45,46].
2. **Compute $\bar{w}$ and $\bar{\theta }$**: Based on the estimated S-CSI, in this phase, we consider Algorithm  1 to compute $\bar{w}$ and $\bar{\theta }$. To better explain the operation of Algorithm 1, its main steps are described as follows:
  I. Randomly generate *L* beamforming vectors at the IRS $\left({\bar{\theta }}_{l},\;l=1,\cdots ,L\right)$. For each ${\bar{\theta }}_{l}$, compute the Maximum-Ratio Transmission (MRT) beamforming vector [47] given by the following:
    (10) $${\bar{w}}_{l}=\frac{\sqrt{P_{t}}{\left[\sqrt{\beta_{c}}{\left(h_{\mathrm{iu}}^{\mathrm{LoS}}\right)}^{H}\theta H_{\mathrm{bi}}^{\mathrm{LoS}}+\sqrt{\beta_{d}}{\left(h_{\mathrm{bu}}^{\mathrm{LoS}}\right)}^{H}\right]}^{H}}{\left||\sqrt{\beta_{c}}{\left(h_{\mathrm{iu}}^{\mathrm{LoS}}\right)}^{H}\theta H_{\mathrm{bi}}^{\mathrm{LoS}}+\sqrt{\beta_{d}}{\left(h_{\mathrm{bu}}^{\mathrm{LoS}}\right)}^{H}|\right|}.$$
    where it is important to highlight that, for a given ${\bar{\theta }}_{l}$, ${\bar{w}}_{l}$ is the optimal transmit beamforming at the BS considering only S-CSI. Here, each individual in the proposed GA is defined as a beamforming vector at the IRS $\left(\bar{\theta }\right)$.
  II. Calculate the fitness defined by (7) of each individual, where SNR at UE is given by the following:
    (11) $$\mathrm{SNR}=\frac{|\left(\sqrt{\beta_{c}}h_{\mathrm{iu}}^{H}\theta H_{\mathrm{bi}}^{\mathrm{LoS}}+\sqrt{\beta_{d}}h_{\mathrm{bu}}^{H}\right)\bar{w}{|}^{2}}{\sigma^{2}},$$
    where $\theta =\mathrm{diag}\left(e^{j{\left[\bar{\theta }\right]}_{1}},\cdots ,e^{j{\left[\bar{\theta }\right]}_{M}}\right)$.
  III. **Elitism:** Select the $N_{f}$ fittest individuals $\left({\bar{\theta }}_{f}\;|\;f=1,\cdots ,N_{f}\right)$, which are preserved without any modification for the next generation.
  IV. **Selection Process:** Select, from the Tournament Method, $\left(L-N_{f}\right)$ parents from the individuals of the current generation.
  V. **Crossover Operator:** With crossover probability $p_{c}$, generate $\left(L-N_{f}\right)$ children from the crossing of the selected parents. Otherwise, with probability $1-p_{c}$, the children are the same as the selected parents. The following individuals are generated: $\left({\bar{\theta }}_{c}\;|\;c=1,\cdots ,L-N_{f}\right)$.
  VI. **Mutation Operator:** With mutation probability $p_{\mathrm{mut}}$, select one child generated in Step V and submit it to the Real Mutation Operator. Otherwise, the selected child is perpetuated for the next generation. This step is run $\left(L-N_{f}\right)$ times. The following individuals are generated $\left({\bar{\theta }}_{p}\;|\;p=1,\cdots ,L-N_{f}\right)$.
  VII. The new population is generated by the union of the individuals generated in Steps III and VI $\left[{\bar{\theta }}_{f},{\bar{\theta }}_{p}\right]$. In other words, the *L* beamforming vectors, $\left({\bar{\theta }}_{l},\;l=1,\cdots ,L\right)$, are updated.
  VIII. Check whether the maximum number of iterations $\left(N_{\mathrm{it}}\right)$ has been reached (stop criterion). If so, return the fittest individual $\left({\bar{w}}_{\mathrm{best}},{\bar{\theta }}_{\mathrm{best}}\right)$. Otherwise, proceed to Step II.
3. **Compute w and θ**: In this phase, the beamforming vectors at BS and the phase shifts at the IRS to be tested are defined at BS and sent to IRS from the controller illustrated in Figure 1. For each beamforming pair, the BS receives feedback of the SNR at UE, and the beamforming pair is evaluated based on (7). This process is repeated for all pairs. In addition, in order to speed up the convergence of the proposed solution and to reduce overhead since we assume a limited mobility scenario, in the process of generating the first population of the proposed method, as illustrated in Figure 2, we consider the knowledge of both beamforming pairs based on S-CSI computed in Phase 2 and the best pair of beamforming computed in the previous channel realization. Note that, in this approach, I-CSI does not need to be estimated. Therefore, we do not need to equip IRS with several RF chains and we do not require any explicit channel estimation processes. The proposed solution is detailed in Algorithm 2, and its main steps are described next.
  I. The first population is generated by the following individuals: (i) (L−2) pairs of beamforming (individuals) randomly generated; (ii) the beamforming pair generated in the first phase $\left(\bar{w},\bar{\theta }\right)$ considering S-CSI; and (iii) the best beamforming pair from the previous channel realization (w,θ).
  II. Compute the fitness of each individual defined by (7), where the SNR at UE is given in (8).
  III. **Elitism:** Select the $N_{f}$ fittest individuals $\left(w_{f},\theta_{f}\;|\;f=1,\cdots ,N_{f}\right)$, which are maintained for the next generation.
  IV. **Selection Process:** Select $(L-N_{f})/2$ individuals from the individuals of the current generation.
  V. **Crossover Operator:** With probability $p_{c}$, generate $(L-N_{f})/2$ children from the crossing of the selected parents. Otherwise, with probability $1-p_{c}$, the children are the selected parents without any modification. The generated individuals are stored in $\left(w_{c},\theta_{c}\;|\;c=1,\cdots ,\left(L-N_{f}\right)/2\right)$.
  VI. **Mutation Operator:** With mutation probability $p_{\mathrm{mut}}$, randomly select an individual selected in Step III. Otherwise, with probability $1-p_{\mathrm{mut}}$, randomly select an individual generated in Step V. In either case, submit the selected individual to the Real Mutation Operator with probability $p_{\mathrm{mut}}$. This step must be run $(L-N_{f})/2$ times, and the following individuals are generated $\left(w_{p},\theta_{p}\;|\;p=1,\cdots ,\left(L-N_{f}\right)/2\right)$.
  VII. The new population is generated by the union of individuals generated in Steps III, V, and VI, $\left(w_{l},\theta_{l}=\left(w_{f},\theta_{f}\right),\left(w_{c},\theta_{c}\right),\left(w_{p},\theta_{p}\right)\;|\;l=1,\ldots ,L\right)$.
  VIII. Check if the maximum number of iterations $\left(N_{\mathrm{it}}\right)$ is reached (stop criterion). If so, then return the fittest individual $\left(w_{\mathrm{best}},\theta_{\mathrm{best}}\right)$. Otherwise, proceed to Step II.

Note that, comparing Algorithms 1 and 2, there are some differences in the form that a new population is created in each generation. In Algorithm 1 the new population (Step VII-Phase 2) is generated following the basic principles of GAs [43]. However, in Algorithm 2, we propose a modification in the Mutation Operator (Step VI-Phase 3) and consequently in the generation of a new population (Step VII-Phase 3). These modifications were made after extensive tests for obtaining maximum performance.

In addition, it should be noted that the proposed solution exploits S-CSI knowledge as an initial point of the beamforming search in order to improve its convergence. With this approach, as we show next, it is possible to reach a close-to-ideal solution with reasonable overhead.
**Algorithm 1:** Algorithm applied in Phase 2 to compute $(\bar{\theta }$, $\bar{w})$.
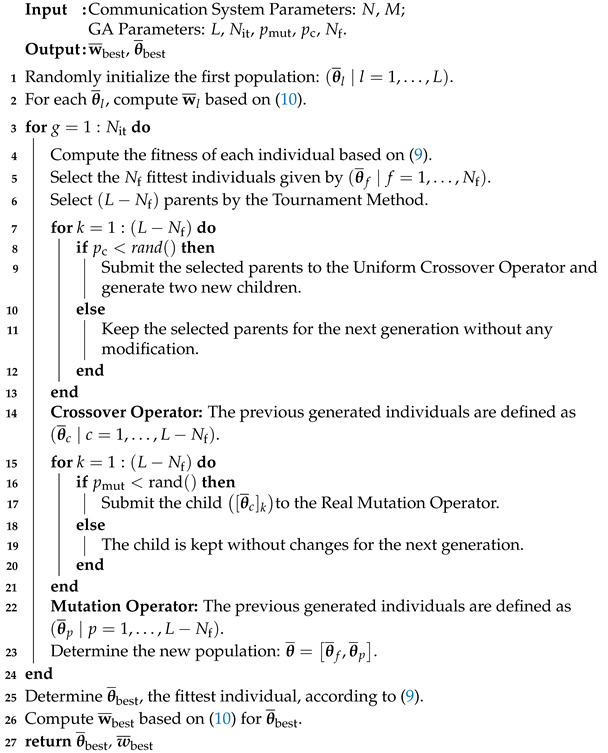


**Algorithm 2:** Algorithm applied in Phase 3 to compute $\left(\theta ,w\right)$.

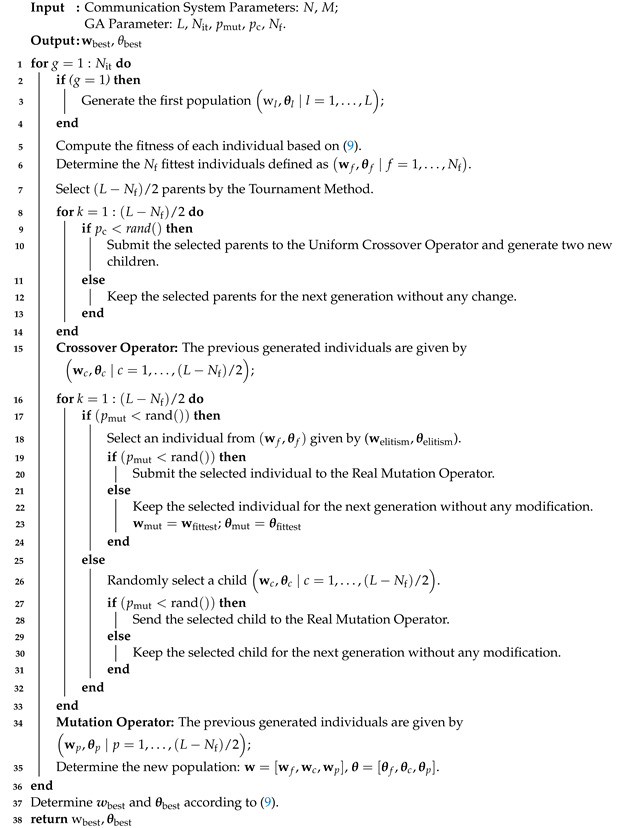



## 5. Simulation Results

**Figure 3:**
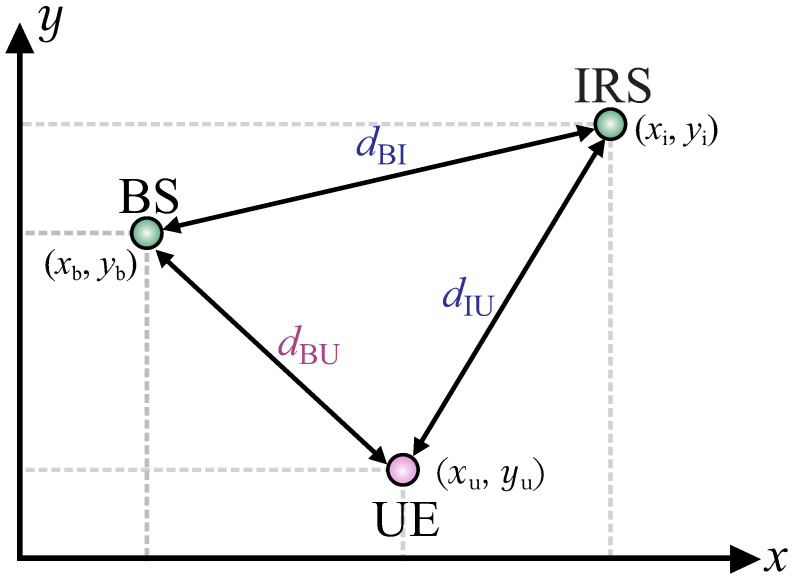
Simulation setup.

In order to show the performance of the proposed solution, we present the results achieved by considering the simulation setup in Figure 3, where $\left(x_{b},y_{b}\right)$, $\left(x_{i},y_{i}\right)$, and $\left(x_{u},y_{u}\right)$ are the BS, IRS, and UE locations, respectively. Therefore, $d_{\mathrm{BI}}$, $d_{\mathrm{BU}}$, and $d_{\mathrm{IU}}$ are the BS-IRS, BS-UE, and IRS-UE distances given by $d_{\mathrm{BI}}=\sqrt{{\left(y_{b}-y_{i}\right)}^{2}+{\left(x_{b}-x_{i}\right)}^{2}}$, $d_{\mathrm{BU}}=\sqrt{{\left(y_{b}-y_{u}\right)}^{2}+{\left(x_{b}-x_{u}\right)}^{2}}$, and $d_{\mathrm{IU}}=\sqrt{{\left(y_{i}-y_{u}\right)}^{2}+{\left(x_{i}-x_{u}\right)}^{2}}$, respectively. The simulation parameters considered in this work are described in Table 2, unless specified otherwise, and all curves present an average of $10^{3}$ different realizations. It is important to highlight that the GA parameters were defined after extensive simulations. Numerical results are shown for three beamforming solutions: (i) *Upper Bound* (the *Upper Bound* benchmark represents the optimal solution to the proposed optimization problem. For this benchmark, we consider perfect knowledge of the I-CSI at the IRS/BS/UE and continuous phase shifts at the IRS elements. In addition, from the I-CSI knowledge, the optimal beamforming at the BS is computed considering Maximum Ratio Transmission (MRT). After this, the proposed optimization problem is converted to a convex problem following the steps described in [8] and the optimal beamforming at the IRS is computed using the CVX toolbox [48]), the optimal beamforming using continuous phase shifts at the IRS, and optimal beamforming at the BS, which is calculated by solving the optimization problem in ([8] Equation (12)) using CVX [48], while the resulting achievable rate is an upper bound for the optimization problem in (9); (ii) *GA-without CSI* (which considers that the beamforming at the BS and at the IRS are computed without any prior channel knowledge at the IRS/BS/UE. More specifically, for this benchmark, we consider discrete phases at the IRS and we compute beamforming at BS and IRS using Algorithm 1 in the manuscript. Here, it is important to hightlight that the *GA-without CSI* differs from the proposed solution based on S-CSI knowledge (*GA-With S-CSI*) by the fact that the first generation of the proposed GA does not consider the beamforming pair computed based on S-CSI as an initial step), the beamforming at the BS, and the phase shifts at the IRS are computed by applying only Algorithm 1, which does not exploit the S-CSI knowledge; and (iii) *GA-with S-CSI*, the proposed solution presented in Section 4, exploiting S-CSI knowledge.

### 5.1. Number of Phase Bits

**Figure 4:**
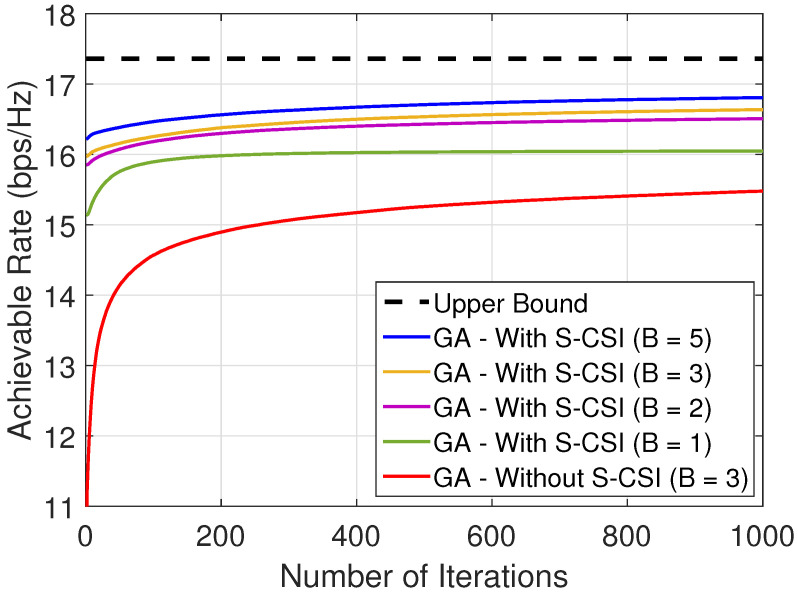
Analysis of the number of controlling bits at each element at the IRS with and without CSI knowledge considering M=40 and $\left(x_{u},y_{u}\right)=\left(95,8\right)$ m, i.e., $d_{\mathrm{IU}}∼5$ m.

In this work, we consider discrete phase shifts at each reflecting element at IRS. As the high resolution (large number of controlling bits *B*) or ideal continuous phase shifts are not energy efficient and are difficult to achieve due to hardware limitations, it is important to achieve a close-to-ideal performance with a small number of controlling bits *B* [49]. Therefore, Figure 4 presents the convergence of difference methods for different values of *B* by considering M=40 and ($x_{u},y_{u}$) = (95, 8) m ($d_{\mathrm{IU}}∼5$ m, i.e., UE close to the IRS). From the results, it can be verified that the proposed solution achieves a suboptimal performance with only B≥2 bits when we consider S-CSI knowledge (Figure 4). From the results, we can conclude that the proposed solution can be applied in practical scenarios with low-resolution phase shifts. Therefore, in the rest of this paper, we consider B=3 bits.

### 5.2. Influence of LoS and Topology

**Figure 5:**
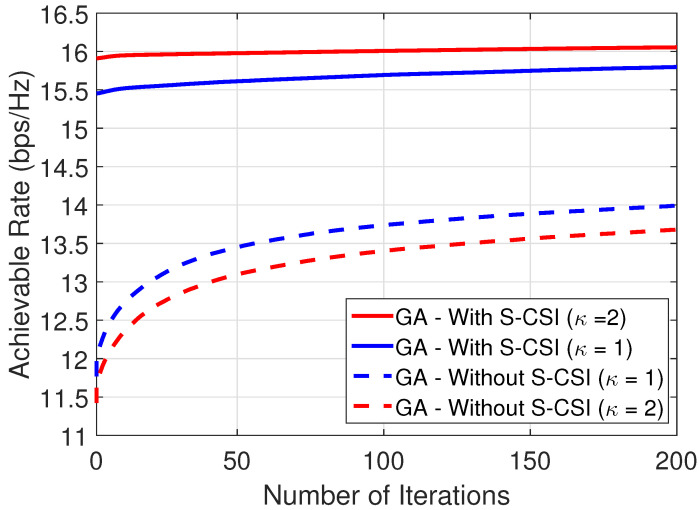
Convergence analysis for M=80 and $\left(x_{u},y_{u}\right)$ = (75, 8) m, i.e., $d_{\mathrm{IU}}∼25$ m.

**Figure 6:**
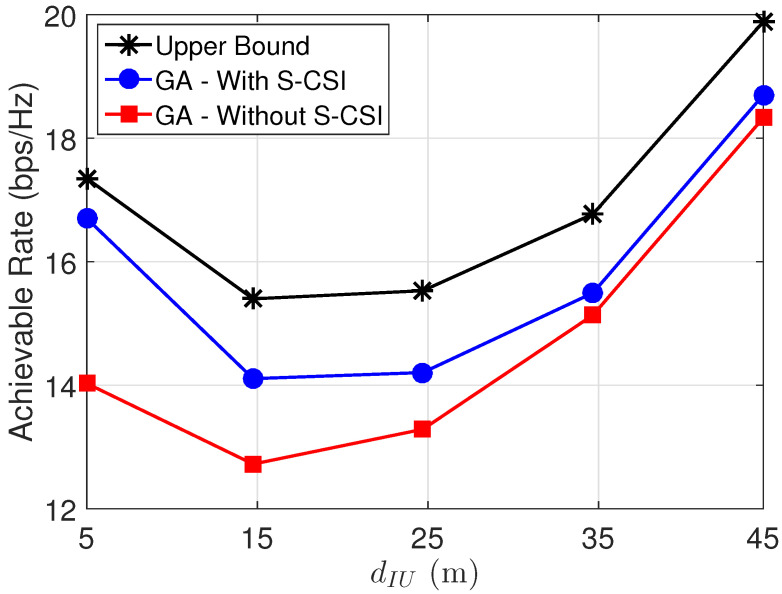
Achievable rate versus BS-UE distance for M=40.

Figure 5 presents the convergence of the proposed solution with and without S-CSI considering κ∈{1,2}. From the results, we can see that the proposed solution considering S-CSI knowledge achieves a better performance with considerably fewer iterations and converges to the suboptimal solution in a few iterations. In addition, we can verify that for a scenario with high influence of the LoS components, the difference between the performance of the proposed solution and the one without S-CSI increases as expected. Figure 6 compares the system’s achievable rate versus BS-UE distance by considering M=40 reflecting elements at the IRS. From the results, it is possible to conclude that the proposed solution achieves a close-to-optimal performance for small values of $d_{\mathrm{IU}}$, i.e, when the user is close to the IRS. Moreover, we also can see that when $d_{\mathrm{IU}}$ decreases, the influence of S-CSI knowledge considerably increases.

**Figure 7:**
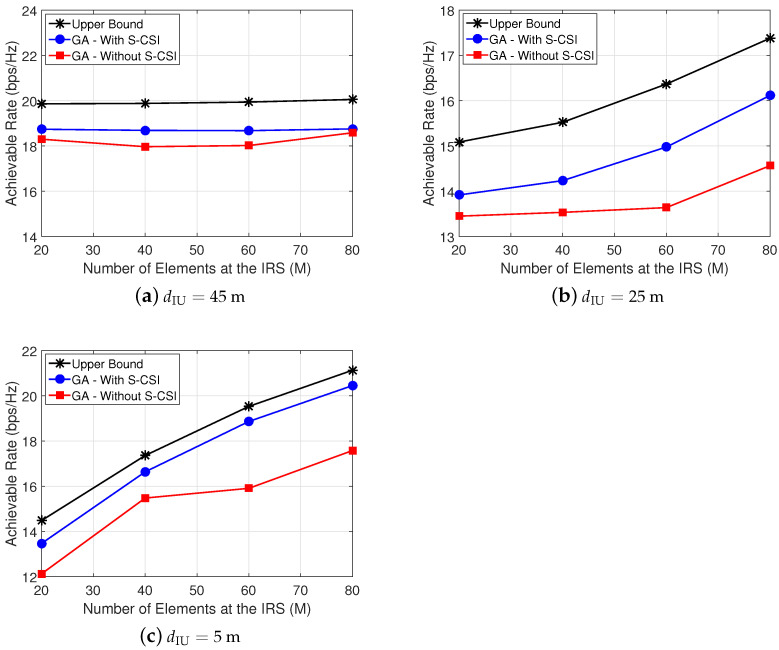
Achievable rate at UE versus the number of reflecting elements (*M*) at IRS.

Figure 7 shows the achievable rate versus the number of elements at the IRS (*M*) for $d_{\mathrm{IU}}\in \left\{45,25,5\right\}$ m. Note the following: (i) for $d_{\mathrm{IU}}=45$ m in Figure 7a, the achievable rate remains almost constant. This can be explained due to the fact that, as UE is far from IRS, increasing *M* does not increase the achievable rate at UE. Thus, in this case, the achievable rate is more dependent on beamforming at the BS; (ii) for $d_{\mathrm{IU}}\leq 25$ m in Figure 7b,c, the achievable rate greatly increases with *M* and the proposed solution reaches a close-to-ideal performance with discrete phases at each IRS element and only a few number of controlling bits. This can be explained due to the fact that, as UE is close to IRS, the UE can explore the IRS gain added to the system. In addition, from the results, we can verify that the position of UE has a considerable influence; this can be related to the gain of the beamforming at BS or at the IRS which the UE can exploit when it is close to the BS or IRS. This conclusion is ratified in Figure 6, where we can note the importance of the UE’s position.

In addition, we can verify from the results presented in Figure 7 that the gap to the upper bound, when we consider S-CSI knowledge, remains constant as *M* increases. However, from the results, we can also verify that, when we do not consider S-CSI knowledge (GA-Without S-CSI), the performance gap in terms of bits per second per Hertz (here defined as $\Delta_{r}$) between *GA-Without S-CSI* and the *Upper Bound* increases as *M* increases, as we can observe in Figure 7b, where for M=20, $\Delta_{r}=∼0.5$ bps/Hz, and for M=80, $\Delta_{r}∼3$ bps/Hz. Therefore, it is possible to verify that, when we consider S-CSI knowledge, the proposed method can maintain the same performance gap independent of IRS’s size, which can be explained due to the fact that, in our proposed solution, we consider the beamforming pair computed based on S-CSI knowledge as an initial solution. Therefore, the first generation of our method is not generated in a random manner, and this increases the quality of the exploitation of the search space by the proposed method and, consequently, increases system performance. Therefore, this also demonstrates the importance of S-CSI knowledge.

### 5.3. Amount of Feedback

**Figure 8:**
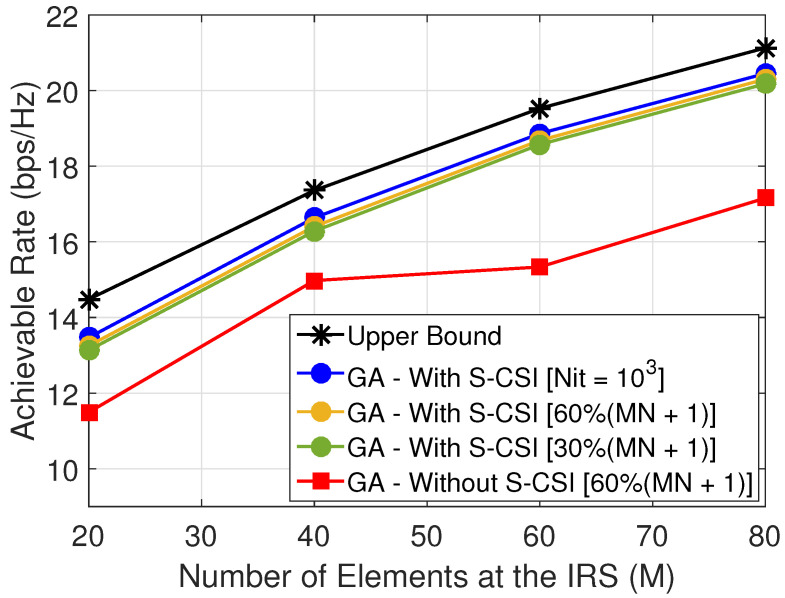
Training overhead of the proposed solution for $\left(x_{u},y_{u}\right)$ = (95,8) m, i.e., $d_{\mathrm{IU}}∼5$ m.

Next we evaluate the amount of feedback from the user to the BS, $N_{\mathrm{it}}L$. If the amount of feedback is not larger than the number of pilots required by a method based on explicit channel estimation, (MN+1) [50], then the proposed solution has a clear advantage. Figure 8 shows the training overhead of our solution in terms of the equivalent number of pilots that would be required for estimating the channel. We can see that the proposed method, even when using only 30% of the training overhead necessary to estimate the channel, performs close to the upper bound. Moreover, recall that our solution does not require additional RF chains at the IRS elements. Finally, it is important to stress that the upper bound considers continuous phases at the IRS and the proposed solution considers a more realistic scenario with discrete phases at the IRS elements. From the results, we can verify that it is possible to obtain a close-to-ideal solution with a reduced amount of feedback from the user.

### 5.4. Imperfect S-CSI Knowledge

The previous results consider perfect S-CSI knowledge. Thus, in order to investigate the robustness of the proposed solution, we next consider the following model for imperfect S-CSI knowledge [51], taking the BS-IRS link as an example:

(12) $${\bar{H}}_{\mathrm{bi}}^{\mathrm{LoS}}=(\sqrt{1-\tau })H_{\mathrm{bi}}^{\mathrm{LoS}}+\sqrt{\tau }E,$$

where $H_{\mathrm{bi}}^{\mathrm{LoS}}$ is the LoS component of the BS-IRS link and ${\bar{H}}_{\mathrm{bi}}^{\mathrm{LoS}}$ represents the imperfect estimation of the LoS component of the BS-IRS link, while E represents the estimation error, for which its entries are i.i.d. zero mean circularly symmetric complex Gaussian random variables with zero mean and unit variance. Moreover, τ∈[0,1] is the estimation accuracy, i.e., if τ=1, there is no correlation between ${\bar{H}}_{\mathrm{bi}}^{\mathrm{LoS}}$ and $H_{\mathrm{bi}}^{\mathrm{LoS}}$; otherwise, if τ=0, we have a perfect S-CSI estimation. We consider that the imperfect estimation of the LoS components or all links (BS-UE and IRS-UE) is generated in the same manner.

**Figure 9:**
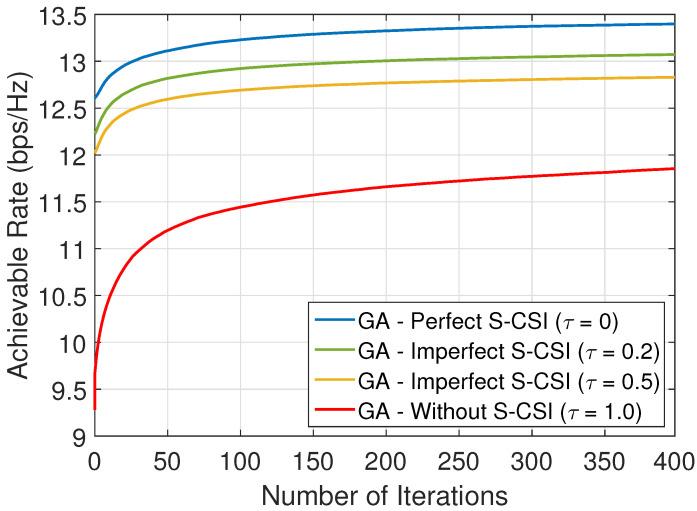
Convergence of the proposed solution with perfect and imperfect S-CSI for M=20 and $\left(x_{u},y_{u}\right)$ = (95,8) m, i.e., $d_{\mathrm{IU}}∼5$ m.

Figure 9 presents the convergence of the proposed solution for different values of τ considering the following schemes: (i) proposed solution with perfect S-CSI; (ii) proposed solution with imperfect S-CSI; and (iii) proposed solution without any CSI. In all cases, we consider κ=1, i.e., both LoS and NLoS components present the same influence on all links. From the results, we can verify that imperfect S-CSI knowledge degrades the performance, but the proposed solution with imperfect S-CSI still achieves a better performance than in the case without S-CSI.

### 5.5. Computational Complexity

In this paper, we proposed a solution based on GA. Therefore, to compute the computational complexity of the proposed solution, it is necessary to consider the complexity of each genetic operator in each phase of the proposed solution. For the second phase, we have the following computation complexity: (i) $O\left(N_{\mathrm{it}}M\right)$ for the Tournament Selection method; and (ii) $O\left(N_{\mathrm{it}}LM\right)$ for the crossover and mutation operators. To finish, in the third phase, we have the following: (i) $O\left(N_{\mathrm{it}}\left(M+N\right)\right)$ for the Tournament Selection method; and (ii) $O\left(N_{\mathrm{it}}L\left(M+N\right)\right)$ for the crossover and mutation operators. Therefore, the computational complexity of the proposed solution is given by $\left[O\left(N_{\mathrm{it}}\left(M+N\right)\left(L+1\right)\right)+O\left(N_{\mathrm{it}}M\left(L+1\right)\right)\right]$, where $N_{\mathrm{it}}$ is the number of generations, *L* is the population size, and *M* and (M+N) is the individual size for the second and third phase, respectively. Therefore, as we can verify, the proposed solution presents a linear computational complexity.

For the importance of computational complexity analysis, for this work, we consider that the issue of training overhead is more relevant as this is one of the main challenges in IRS-assisted communication systems. Therefore, in the manuscript, we evaluate the amount of feedback from the user to the BS, $N_{\mathrm{it}}L$ (Figure 8). If the amount of feedback is not larger than the number of pilots required by a method based on explicit channel estimation, (MN+1) [50], then the proposed solution has a clear advantage. As we can observe from the numerical results in Figure 8, the proposed method, even when using only 30% of the training overhead necessary to estimate the channel, performs close to the upper bound.

## 6. Conclusions

In this paper, we evaluated two different solutions based on GA in order to design beamforming at BS and IRS, with a maximum transmit power constraint and discrete phase shifts at IRS. First, we proposed a solution that does not consider any knowledge of the CSI at BS, and a suboptimum design of the beamforming at BS and IRS was performed. Next, we proposed another solution to solve the beamforming problem only by exploiting S-CSI knowledge. The novel solutions were evaluated considering different setups, and from the results, we can conclude that they achieve a great performance considering discrete phases with a few numbers of controlling bits at each element and with a reasonable amount of feedback from UE. This shows that the proposed solutions are very attractive in practice. As future works, we intend to investigate the system overhead in a MIMO multi-user system.

## Figures and Tables

**Table 1:** The meaning of the main GA parameters.

GA Parameter	Meaning
Gene	An element of the beamforming vector
Individual	The set of optimization variables in the problem
Population	A set of solutions for the optimization problem
Parents	Individuals selected in the Selection Method
Children	Individual generated by the Crossover Operator
Fitness Function	Metric defined by the optimization problem
Fitness	Output of the fitness function
Generation	Iteration of the algorithm

**Table 2:** Simulation parameters.

Parameter	Value
*N*	10
$M_{v}$	10
$N_{\mathrm{it}}$	$10^{3}$
$N_{f}$	2
$N_{\mathrm{tourn}}$	2
$p_{\mathrm{mut}}$	8% (case with S-CSI)
$p_{\mathrm{mut}}$	5% (case without S-CSI)
$p_{c}$	90% (for both cases)
$d_{\mathrm{IRS}}$	0.5
$G_{t}$	3 dBi
$G_{r}$	3 dBi
$f_{c}$	4 GHz
$d_{\mathrm{BS}}$	λ/2
κ	1 (if not specified otherwise)
$\left(x_{b},y_{b}\right)$	(50, 10) m
$\left(x_{i},y_{i}\right)$	(100, 10) m
$\sigma^{2}$ [9]	−80 dBm
$P_{t}$	20 dBm
$\beta_{0}$	41.98
$\beta_{1}$	21.98

## Data Availability

Not applicable.

## Abbreviations

BS	Base Station
CSI	Channel State Information
GA	Genetic Algorithm
I-CSI	Instantaneous CSI
IRS	Intelligent Reflecting Surfaces
LoS	Line-of-Sight
NLoS	Non-LoS
MIMO	Multiple-Input Multiple-Output
MISO	Multiple-Input Single-Output
mMIMO	massive MIMO
MRC	Maximum Ratio Combining
PSO	Particle Swarm Optimization
S-CSI	Statistical CSI
SNR	Signal-to-Noise Ration
UE	User Equipment
ULA	Uniform Linear Array
UPA	Uniform Planar Array
$\beta_{0},\;\beta_{1}$	Constant path loss factors.
$\beta_{c}$	Pathloss of the BS-UE link
$\beta_{d}$	Pathloss of the BS-IRS-UE link
θ	Phase shift vector at the IRS
$\bar{\theta }$	Phase shift vector at the IRS considering only S-CSI
$\left(\theta_{\mathrm{IRS}},\theta_{\mathrm{BS}}\right)$	Elevation angles
κ	Rician factor for all links
λ	Wavelength
ξ	Amplitude reflection coefficient
σ	Power noise
τ	Estimation accuracy
$\phi_{\mathrm{bi}}$	Random phase in the LoS components of the BS-IRS link
$\phi_{\mathrm{bu}}$	Random phase in the LoS components of the BS-UE link
$\phi_{\mathrm{iu}}$	Random phase in the LoS components of the IRS-UE link
$\varphi_{\mathrm{IRS}}$	Azimuth angle
*B*	Number of bits per each element at the IRS
$d_{\mathrm{bi}}$	BS-IRS link distance
$d_{\mathrm{BS}}$	Distance among the BS elements
$d_{\mathrm{bu}}$	BS-UE link distance
$d_{\mathrm{IRS}}$	Distance among the IRS elements
$d_{\mathrm{iu}}$	IRS-UE link distance
E	Estimation error
$G_{r}$	Antenna gain at the UE
$G_{t}$	Antenna gain at the BS
$h_{\mathrm{bu}}$	BS-UE channel vector
$h_{\mathrm{bu}}^{\mathrm{LoS}}$	Deterministic LoS components of the BS-UE link
$h_{\mathrm{bu}}^{\mathrm{NLoS}}$	Rayleigh fading of the BS-UE link
$h_{\mathrm{iu}}$	IRS-UE channel vector
$h_{\mathrm{iu}}^{\mathrm{LoS}}$	Deterministic LoS components of the IRS-UE link
$h_{\mathrm{iu}}^{\mathrm{NLoS}}$	Rayleigh fading of the IRS-UE link
$H_{\mathrm{bi}}$	BS-IRS channel matrix
$H_{\mathrm{bi}}^{\mathrm{LoS}}$	Deterministic LoS components of the BS-IRS link
${\bar{H}}_{\mathrm{bi}}^{\mathrm{LoS}}$	Imperfect estimation of the LoS components of the BS-IRS link
*K*	Phase shift levels
*L*	GA population size
*M*	Number of elements at the IRS
$M_{h}$	Number of elements in the horizontal level at the IRS
$M_{v}$	Number of elements in the vertical level at the IRS
*n*	Additive white Gaussian noise at the UE
*N*	Number of antennas at the BS
$N_{\mathrm{it}}$	Number of iterations at the GA
$N_{f}$	Number of elements selected by the elitism process
$p_{c}$	Crossover probability
$p_{\mathrm{mut}}$	Mutation probability
$P_{t}$	Transmit power at the BS
R	Achievable rate at the UE
*s*	Transmitted data
T	Set of all possible discrete phases at the IRS
$T_{\mathrm{tourn}}$	Number of elements selected to compete in a tournament.
w	Beamforming vector at the BS
$\bar{w}$	Beamforming vector at the BS considering only S-CSI
$\left(x_{b},y_{b}\right)$	BS position
$\left(x_{i},y_{i}\right)$	IRS position
$\left(x_{u},y_{u}\right)$	UE position
*y*	Received signal at the IRS
